# Associations between Burkitt Lymphoma among Children in Malawi and Infection with HIV, EBV and Malaria: Results from a Case-Control Study

**DOI:** 10.1371/journal.pone.0002505

**Published:** 2008-06-18

**Authors:** Nora Mutalima, Elizabeth Molyneux, Harold Jaffe, Steve Kamiza, Eric Borgstein, Nyengo Mkandawire, George Liomba, Mkume Batumba, Dimitrios Lagos, Fiona Gratrix, Chris Boshoff, Delphine Casabonne, Lucy M. Carpenter, Robert Newton

**Affiliations:** 1 Department of Public Health, University of Oxford, Oxford, United Kingdom; 2 Department of Pediatrics, College of Medicine, Blantyre, Malawi; 3 Department of Histopathology, College of Medicine, Blantyre, Malawi; 4 Department of Surgery, College of Medicine, Blantyre, Malawi; 5 Department of Ophthalmology, College of Medicine, Blantyre, Malawi; 6 Wolfson Institute for Biomedical Research based at University College London, London, United Kingdom; 7 Cancer Epidemiology Unit, University of Oxford, Oxford, United Kingdom; 8 Epidemiology and Genetics Unit, Department of Health Sciences, University of York, York, United Kingdom; London School of Hygiene & Tropical Medicine, United Kingdom

## Abstract

**Background:**

Burkitt lymphoma, a childhood cancer common in parts of sub-Saharan Africa, has been associated with Epstein Barr Virus (EBV) and malaria, but its association with human immunodeficiency virus (HIV) is not clear.

**Methodology/Principal Findings:**

We conducted a case-control study of Burkitt lymphoma among children (aged ≤15 years) admitted to the pediatric oncology unit in Blantyre, Malawi between July 2005 and July 2006. Cases were 148 children diagnosed with Burkitt lymphoma and controls were 104 children admitted with non-malignant conditions or cancers other than hematological malignancies and Kaposi sarcoma. Interviews were conducted and serological samples tested for antibodies against HIV, EBV and malaria. Odds ratios for Burkitt lymphoma were estimated using unconditional logistic regression adjusting for sex, age, and residential district. Cases had a mean age of 7.1 years and 60% were male. Cases were more likely than controls to be HIV positive (Odds ratio (OR))  = 12.4, 95% Confidence Interval (CI) 1.3 to 116.2, *p* = 0.03). ORs for Burkitt lymphoma increased with increasing antibody titers against EBV (*p* = 0.001) and malaria (*p* = 0.01). Among HIV negative participants, cases were thirteen times more likely than controls to have raised levels of both EBV and malaria antibodies (OR = 13.2; 95% CI 3.8 to 46.6; *p* = 0.001). Reported use of mosquito nets was associated with a lower risk of Burkitt lymphoma (OR =  0.2, 95% CI, 0.03 to 0.9, *p* = 0.04).

**Conclusions:**

Our findings support prior evidence that EBV and malaria act jointly in the pathogenesis of Burkitt lymphoma, suggesting that malaria prevention may decrease the risk of Burkitt lymphoma. HIV may also play a role in the etiology of this childhood tumor.

## Introduction

Endemic Burkitt lymphoma is a common childhood cancer in parts of sub-Saharan Africa, including Malawi [1], [2] with a male preponderance and peak age of tumor occurrence at 7 years [3], [4]. Jaw tumors, usually involving multiple quadrants, are a characteristic feature, especially in younger children [5]. Two infectious agents, EBV and *Plasmodium falciparum* malaria have been associated with the etiology of Burkitt lymphoma. At least 90% of endemic Burkitt lymphoma cases are thought to be EBV-associated [6], [7], with supportive evidence including the presence of EBV-DNA clonally integrated into tumor tissue and sero-epidemiological associations with EBV antibodies [7]–[9]. Ecological studies demonstrating a positive association between Burkitt lymphoma and malaria [10]–[12] have only recently been supported by a case-control study in Uganda [13]. Malaria is the leading cause of death in children under 5 years of age in Malawi. Sixty-eighty percent of infants living alongside Lake Malawi were found to be infected with *Plasmodium falciparum* by the age of 10 months [14]. Children under the age of five years had suffered an average of nine clinical episodes of malaria illness per year and many remain chronically parasitaemic year-round [14]. Use of insecticide-treated nets is one of the main objectives for the Roll Back Malaria campaign in Malawi and other malaria-endemic countries [15].

Epidemiological evidence for an association between HIV and endemic Burkitt lymphoma is more uncertain [16], [17]. While one case-control study from Uganda reported a positive association with HIV infection [18]; a small subset of preliminary data from this study reported no association [19]. Associations with socio-economic and environmental factors have also been suggested but relatively little studied [5], [20]. Here, we report risk factors for childhood Burkitt lymphoma from a case-control study conducted in Malawi focusing particular attention on three infections: HIV, EBV and malaria.

## Materials and Methods

**Figure 1:**
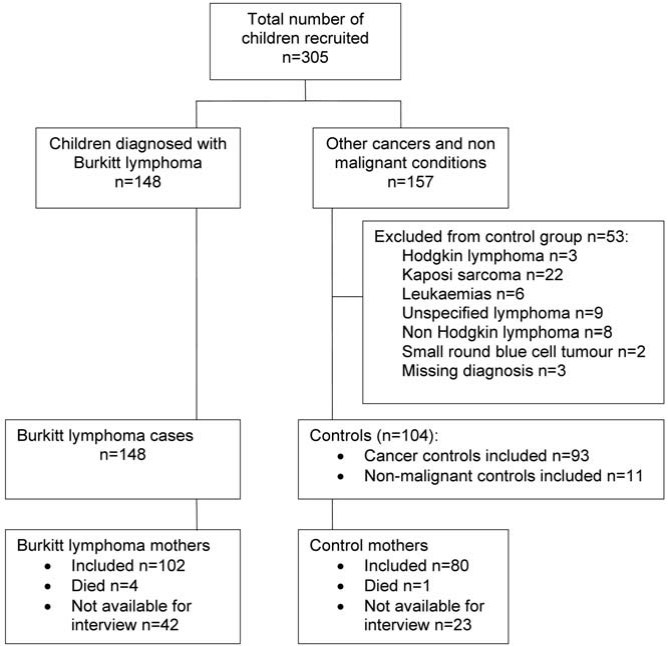
Burkitt lymphoma cases and controls recruitment flow chart.

This case-control study was nested within a larger study of childhood cancer conducted at the main hospital in Blantyre, Malawi, between July 2005 and July 2006. All 305 children aged 15 years or younger with a provisional diagnosis of cancer admitted to the pediatric oncology ward in Blantyre were recruited into the larger study. Preliminary clinical diagnoses of cancer were made by one investigator (EM) and were confirmed by histology, cytology or other laboratory investigations where possible. The study included 148 cases, 109 (75%) of whom were histologically diagnosed with Burkitt lymphoma, and 104 controls, 73 (74%) of whom were histologically diagnosed with other conditions (Figure 1). Just over half (n = 83) of children with Burkitt lymphoma presented with jaw lesions, although abdominal lesions were also relatively frequent (n = 57). Controls included 93 children diagnosed with cancer: Wilms tumor (n = 30), retinoblastoma (n = 18), rhabdomyosarcoma (n = 18), non-malignant (non-infectious) conditions (n = 11), neuroblastoma (n = 6), hepatocellular carcinoma (n = 4), malignant teratoma (n = 4), bone tumor (n = 4), ovarian tumor (n = 3), and one of six other tumor types/sites (abdominal, brain, hepatoblastoma, soft tissue tumor, squamous cell carcinoma and yolk sac tumor). A further 11 controls were children with non-malignant conditions, admitted to the oncology ward with a provisional diagnosis of cancer, who were subsequently found to have non-malignant conditions (aplastic anaemia, benign cystic teratoma, benign mesenchymal tumor, cellular mesoblastic nephroma, chronic inflammation, Down's syndrome, hemangioma, liver abscess, melanotic neuroectodermal tumor of infancy, ranula). All children diagnosed with hematological cancers (including leukemia, non-Burkitt lymphoma, and small round blue cell tumors) and Kaposi's sarcoma were excluded from the control group, because of possible diagnostic overlap and previously known associations with HIV.

### Recruitment and data collection

Five local nurses were employed and trained to recruit children and their mothers into the study and to administer standardized questionnaires. The parent or guardian of each child was approached and invited to participate in the study and provide written informed consent for their child to be included in the study. All children seen in the pediatric oncology ward with suspected cancer were routinely tested for HIV infection. The mother of each child was also asked to consent to be interviewed and also provided a serologic sample for testing for antibodies against HIV, EBV, malaria and syphilis. Appropriately trained staff provided pre- and post-HIV test counseling. Mothers of 102 (69%) cases and of 80 (77%) controls consented and were interviewed about demographic and socio-economic factors, their child's birth, household characteristics and their own sexual and reproductive histories. Ethical approval for the study was obtained from the Oxford Tropical Research Ethics Committee and the Malawian College of Medicine Research and Ethics Committee.

### Laboratory procedures

HIV serostatus was determined on site for 146 cases (99%) and 93 controls (90%) and also for 97 (95%) mothers of cases and 75 (94%) mothers of controls using Determine HIV (Abbott Laboratories, Illinois, USA) as a screening test and Uni-Gold™ HIV (Trinity Biotech PLC, Ireland) as a confirmatory test, as used in other studies from Malawi [16], [21]. Serological testing for IgG viral capsid antigen EBV antibodies was performed for 138 (93%) cases, 95 (91%) controls, 100 (98%) mothers of cases and for 77 (96%) mothers of controls, at the Wolfson Institute for Biomedical Research based at University College, London using indirect immunofluorescence (IFA) [22]. This well validated serological test has been used elsewhere [23]. For the purposes of this analysis, children were categorized into one of three EBV antibody groups: low (antibodies levels of 1∶640 or less), medium (antibodies levels of 1∶1280 to 1∶2560), and high (antibody levels of 1∶5120 to 1∶20480). Because the number of mothers was smaller, they were categorized into two EBV antibody groups: low (antibody levels of 1∶640 or less) and medium/high (antibody levels of 1∶1280 to 1∶20480). Antibodies against *Plasmodium falciparum* were measured for 139 (94%) cases and 96 (92%) controls and also for 100 (98%) mothers of cases and 77 (96%) mothers of controls, at the Centre for Clinical Vaccinology and Tropical Medicine in Oxford using an Enzyme-linked ImmunoSorbent Assay (ELISA) [24]. The technique used a *Plasmodium falciparum* schizont extract (PfSE) as the malaria antigen, which has been used elsewhere [25]. Using optical density (OD) readings as a surrogate measure of malaria antibody titers, children were categorized as either negative/low (OD<0.2), or medium/high (OD≥0.2) and mothers as either low (OD<1) or medium/high (OD≥1).

In addition, syphilis tests were conducted in Malawi, for 99 (97%) mothers of cases and for 77 (96%) mothers of controls, using the Rapid Plasma Reagin (RPR) test (Abbot Determine, Alloa, Scotland) and, if positive, confirmed by the *Treponema pallidum* hemaglutination assay (TPHA) (Biotech TPHA kit, Biotech Laboratories Ltd, Suffolk, UK). Both syphilis tests were conducted according to the manufacturer's instructions. Women were defined as having current or prior syphilis infection if they had positive RPR and TPHA tests.

### Statistical analyses

Data were entered into an Access database in Malawi [26] and analyses were performed in Oxford using STATA computer software (version 9) [27]. Odds ratios (ORs) were estimated by maximum likelihood using unconditional logistic regression. All ORs relating to data on children were adjusted for child's age (under 5 years, and 5 years and over), sex and residence (rural, urban), while those relating to data on mothers were adjusted for mother's age (under 30 years, 30 years and over) and residence. Analyses examining associations with EBV and malaria were restricted to HIV negative cases and controls to exclude possible confounding effects of HIV on these antibody titers. All *p* values reported were obtained using two-sided tests of statistical significance.

To establish an index of poverty, data on eight proxy markers were combined (ownership of a phone, television, fridge, radio, chickens, and availability of piped water, ownership of land and electricity in the home). Individuals were assigned to one of two groups: those ‘better off’, and those ‘worse off’. The ‘better off’ group were those having either a television, fridge, phone, electricity or piped water, in addition to one or more of the other three markers listed above, while the ‘worse off’ group did not posses any of these five items).

## Results

### General characteristics

Sixty percent of all 148 cases were boys and their ages ranged from 2 years to 15 years (mean age = 7.1 (standard deviation (SD) 2.6 years). Controls were younger (mean age  =  5.1 (SD 3.9 years) and included a slightly smaller percentage of boys (55%) (Table 1). Almost 80% (n = 111) of cases and 70% (n = 69) of controls were reported to reside in rural areas and the percentage living in “better off” households was lower among cases (38% versus 49%). Only four cases and one control reported having a mother who had died. Comparison of characteristics of the 135 HIV negative cases and 90 HIV negative controls showed similar patterns (Table 1).

**Table 1:** Characteristics of children diagnosed with Burkitt lymphoma (cases) and their controls.

CHARACTERISTIC		All children		HIV negative children	
		Cases (n = 148)	Controls (n = 104)	Cases (n = 137)	Controls (n = 91)
1 ***Age*** * (years)*	Median (IQR)	7 (5 to 9)	4 (2 to 8)	7 (5 to 9)	4 (2 to 7)
	Mean (SD)	7.1 (2.6)	5.1 (3.9)	7.2 (2.6)	5.1 (3.8)
***Age groups***	0 to 4	19 (13%)	57 (55%)	15 (11%)	49 (54%)
	5 to 15	126 (87%)	46 (45%)	120 (89%)	41 (46%)
***Sex***	Male	89 (60%)	57 (55%)	81 (59%)	53 (58%)
	Female	59 (40%)	47 (45%)	56 (41%)	38 (42%)
1 ***Residence***	Rural	111 (78%)	69 (70%)	104 (79%)	66 (74%)
	Urban	32 (22%)	30 (30%)	28 (21%)	23 (26%)
2 ***Markers of poverty***	Worse off	63 (62%)	41 (51%)	99 (72%)	57 (63%)
	Better off	39 (38%)	39 (49%)	38 (28%)	34 (37%)
1 ***Child birth order***	Less than four	100 (68%	66 (65%)	91 (67%)	58 (65%)
	Four or higher	46 (32%)	36 (35%)	44 (33%)	31 (35%)
1 ***Mother alive***	Yes	144 (97%)	103 (99%)	135 (99%)	90 (99%)
	No	4 (3%)	1 (1%)	2 (1%)	1 (1%)

1 Numbers may not add to the total because of missing values.

2 See methods.

### HIV, EBV and malaria

The percentage of HIV positive children was small for both cases and controls (6% (n = 9) for cases and 2% (n = 2) for controls). However, after adjustment for child's age, sex and residence, cases were 12 times more likely than controls to be HIV positive (OR = 12.4, 95% CI 1.3 to 116.2, *p* = 0.03) (Table 2). When analyses were restricted to cases (n = 109) and controls (n = 65) whose diagnoses were histologically verified, cases were still almost 10 times more likely than controls to be HIV positive (OR = 9.7, 95% CI 1 to 95.7, *p* = 0.02).

**Table 2:** Association between antibodies against HIV, EBV and malaria, in relation to risk of Burkitt lymphoma.

RISK FACTOR	Prevalence			Number		1Adjusted OR (95% CI)
						χ^2^ for heterogeneity
ALL CHILDREN						
		Cases	Controls	Cases	Controls	
* ***Child HIV status***	Positive	6%	2%	9	2	12.4 (1.3 to 116.2)
	Negative	94%	98%	137	91	1.0
						χ^2^ (1df) = 7.2 *p* = 0.03
**HIV NEGATIVE ONLY**						
* 2 ***EBV antibodies***	Low	9%	43%	12	38	1.0
	Medium	31%	36%	39	32	4.1 (1.6 to 10.1)
	High	60%	21%	77	19	14.8 (5.8 to 38.5)
						χ^2^ (1df) = 37.8 *p_trend_*<0.001
* 3 ***Malaria antibodies***	Negative/Low	29%	53%	38	48	1.0
	Medium/High	71%	47%	91	42	2.4 (1.2 to 4.4)
						χ^2^ (1df) = 6.9 *p*<0.01

1 Adjusted for child age, sex and residence.

2 EBV antibody categories: Low (≤1∶640), Medium (1∶1280 to 1∶2560), High (1∶5120 to 1∶20480).

3 Malaria antibody categories: Negative/low (OD<0.2), Medium/high (OD≥0.2).

* Data missing.

Sixty percent (n = 77) of HIV negative cases and 21% (n = 19) of HIV negative controls had high EBV antibody levels. ORs for developing Burkitt lymphoma were strongly associated with increasing titers of antibodies against EBV antigens, increasing from 4.1 for those with medium levels, to 14.8 for those with high levels, as compared to those with the lowest levels (*p*_trend_<0.001) (Table 2). Seventy-one percent (n = 91) of HIV negative cases had medium/high levels of malaria antibodies compared to 47% (n = 42) of HIV negative controls. The adjusted OR for developing Burkitt lymphoma was 2.4 for those whose antibodies against malaria were medium to high compared to those whose antibodies were negative or low (*p* = 0.01) (Table 2).

The joint effects of EBV and malaria antibodies were examined by estimating adjusted ORs for children with raised levels of EBV antibodies only, raised levels of malaria antibodies only, and raised levels of both EBV and malaria antibodies, compared with children with low levels of both (Table 3). ORs were 1.4 for children with raised EBV antibody titers only and 5.7 for those with raised malaria antibody titers only, while the OR for children with high levels of both EBV and malaria antibodies was estimated to be 13.2 (95% CI 3.8 to 46.6, *p*<0.001). Although the data were limited, there was no difference in EBV and malaria antibody levels between HIV negative and positive Burkitt lymphoma cases (data not shown).

**Table 3:** Joint effects of serological results for EBV and malaria in HIV negative cases and controls

			Cases/Controls (126/89)	1Adjusted OR (95% CI)
2EBV antibodies		3Malaria antibodies		
	Low	Negative/Low	5/22	1.0
		Medium/High	7/16	1.4 (0.3 to 6.3)
	Medium/High	Negative/Low	32/25	5.7 (1.6 to 20.7)
		Medium/High	82/26	13.2 (3.8 to 46.6)
				χ^2^ (1df) = 31 *p*<0.001

1 Adjusted for child age, sex and residence

2 EBV antibody categories: Low (≤1∶640), Medium/High (≥1∶1280)

3 Malaria antibody categories: Negative/low (OD<0.2), Medium/high (OD≥0.2)

### Mosquito net use

Questions regarding household mosquito net ownership and use, were introduced eight months after the study began and were, therefore, only available for 30 (20%) of Burkitt lymphoma cases and 25 (24%) of controls (Table 4). Eighty percent (n = 20) of control households and 60% (n = 18) of Burkitt lymphoma households reported owning mosquito nets (OR = 0.2, 95% CI 0.03 to 1.2, *p* = 0.08), while 79% (n = 20) of controls and 57% (n = 17) of cases reported having used them (OR = 0.2, 95% CI 0.03 to 0.9, *p* = 0.04).

**Table 4:** Use of mosquito nets in Burkitt lymphoma cases and controls.

RISK FACTOR		Cases	Controls	1Adjusted OR (95% CI)
		n = 30	n = 25	χ^2^ for heterogeneity
**ALL CHILDREN**				
2 ***Home mosquito nets ownership***				
	Yes	18	20	0.2 (0.03 to 1.2)
	No	12	5	1.0
				χ^2^ (1df) = 3.7 *p* = 0.08
2 ***Child use mosquito net?***				
	Yes	17	20	0.2 (0.03 to 0.9)
	No	12	5	1.0
				χ^2^ (1df) = 5.0 *p* = 0.04

1 Adjusted for child age, sex and residence.

2 Numbers reduced towards end of study because questions introduced later.

### Maternal characteristics

Characteristics of 80 HIV negative cases and controls are shown in Table 5. OR estimates for Burkitt lymphoma were almost 5 fold higher for children who had more than 3 siblings alive than those with up to 3 siblings alive (OR = 4.5, 95% CI 1.4 to 14.1, *p* = 0.01). Although not statistically significant, 19% (n = 15) of mothers of cases and 7% (n = 4) of mothers of controls reported having children with different biological fathers (OR = 2.5, 95% CI 0.8 to 8.5, *p* = 0.1). The OR was raised for children whose mothers reported more than 1 lifetime sexual partner (adjusted OR = 3.0, 95% CI 1.2 to 7.6, *p* = 0.02). Seventy-nine percent (n = 65) of case mothers and 70% (n = 40) of control mothers had high EBV antibody titers (OR =  2.1, 95% CI 0.8 to 5.7, *p* = 0.1). Maternal syphilis status and level of malaria antibodies were not associated with Burkitt lymphoma in the child.

**Table 5:** Odds ratio estimates for maternal sexual and reproductive factors and serological test results, restricted to HIV negative mothers.

RISK FACTOR		Cases/Controls	1Adjusted OR (95%CI)
			χ^2^for heterogeneity
**HIV NEGATIVE MOTHERS OF HIV NEGATIVE CHILDREN**			
**INTERVIEWS**			
***Number of children alive***			
	Less than four	26/30	1.0
	Four or more	54/26	4.5 (1.4 to 14.1)
			χ^2^ (1df) = 7.4 *p* = 0.01
***Children with different fathers***			
	No	65/53	1.0
	Yes	15/4	2.5 (0.8 to 8.5)
			χ^2^ (1df) = 2.2 *p* = 0.1
***Reported number of sexual partners***			
	Less than two	37/39	1.0
	Two or more	38/15	3.0 (1.2 to 7.6)
			χ^2^ (1df) = 5.8 *p* = 0.02
**SEROLOGICAL TESTS**			
2 ***Mother malaria antibodies***			
	Low	32/28	1.0
	High	50/29	1.2 (0.5 to 2.7)
			χ^2^ (1df) = 0.2 *p* = 0.6
3 ***Mother EBV***			
	Low	17/17	1.0
	Medium/High	65/40	2.1 (0.8 to 5.7)
			χ^2^ (1df) = 2.3 *p* = 0.1
***Syphilis***			
	Negative	71/52	1.0
	Positive	11/6	1.2 (0.4 to 3.8)
			χ^2^ (1df) = 0.1 *p* = 0.8

1 Adjusted for mother age and residence.

2 Mother malaria antibody categories: Low (OD<1), Medium/high (OD≥1).

3 EBV antibody categories: Low (≤1∶640), Medium/high (1∶1280 to 1∶20480).

## Discussion

This case-control study of childhood Burkitt lymphoma in Malawi has focused on the potential role played by three different infectious diseases in children: HIV, EBV and malaria. The positive association between Burkitt lymphoma and HIV found here is similar to that reported from a recent study conducted in Uganda [18]. Most other reports addressing this issue in African children have been case series or based on routinely collected data and have not been able to adjust for important confounding factors such as child's age, sex and residence [16]. HIV may be acting as an indirect co-factor in the etiology of Burkitt lymphoma by reducing the effectiveness of T cell based immune response to oncogenic viruses [28] or by reducing the EBV-specific T cell function, leading to proliferation of EBV infected B cells and eventual tumor formation [29]. These findings suggest that reducing HIV incidence, or treatment of HIV with antiretroviral drugs, may lead to a reduction in the incidence of Burkitt lymphoma in children. However, the number of HIV positive cases of Burkitt lymphoma reported to date is relatively small and considerable uncertainty therefore remains about the magnitude of association between HIV and Burkitt lymphoma among African children.

In accordance with results from previous epidemiological studies from sub-Saharan Africa, we have clear evidence to suggest that the risk of Burkitt lymphoma increases with increasing titers of antibodies against EBV in the child [7], [9], [13]. Because Burkitt lymphoma is a systemic disease and because we have used a case-control design it is possible that the raised antibody titers against EBV were a result of the tumor rather than the cause of the disease (reverse causality). Nevertheless, the results of our case-control study are similar to those from a prospective investigation [7], [9].

In contrast, previous evidence for an association between Burkitt lymphoma and malaria has been largely ecological [30]–[32]. This case-control study also provides more direct evidence of an association between malaria antibodies in the child and risk of Burkitt lymphoma comparable to those recently reported from a similar study in Uganda [13]. While anti-malarial antibodies are not thought to reflect current infection, but rather previous exposure, uncertainty still remains about the longevity of these antibodies post-infection [33]–[36].

There is clear evidence from clinical trials that use of bed nets reduces mortality from malaria among children [37], [38] and our findings raise the possibility that this preventive measure against malaria may have potential to decrease the risk of Burkitt lymphoma in African children. Reported use of household insecticides and mosquito nets were associated with a lower risk of Burkitt lymphoma in Uganda, lending support to the view that malaria prevention may impact on the incidence of this childhood cancer [13]. However, it is important to note that our results were based on a small subset of cases and controls and could be explained by the effects of residual confounding by socio-economic status. In Malawi, widespread distribution of subsidized bed nets began in 2002, although the prevalence of use has been reported to be higher in urban households compared to those in rural areas [39]. No decline in the frequency of Burkitt lymphoma has yet been reported.

In accordance with the Uganda case-control study [13], we also found evidence to suggest that EBV and malaria act jointly as risk factors for Burkitt lymphoma. Compared with those who had low levels of both EBV and malaria antibodies, our data suggest that children with high levels of both antibodies have 13 times the risk of developing the tumor. While EBV viral load in blood has been previously reported to be highest in children from malaria endemic areas [40], the specific nature of the relationship between EBV and malaria is unclear. Two hypotheses have been suggested to explain this. First, malaria leads to reactivation and proliferation of EBV latently infected B cells and second, that malaria may also lead to suppression of EBV-specific T cell immunity, which allows EBV to proliferate [41]–[43]. The apparently synergistic effect suggests that infection with both EBV and malaria may be needed to facilitate tumor formation.

That the odds of Burkitt lymphoma increased with the number of children alive reported by mothers may be linked to exposure of the index child to EBV infection and re-infection from other family members [44], [45]. Further, the odds of Burkitt lymphoma increased significantly with increasing number of self-reported maternal lifetime sexual partners and an association with high EBV antibody titers in the mother was suggested. Although based on small numbers, these findings may suggest that mothers of Burkitt lymphoma cases are exposed to EBV (or perhaps multiple strains of EBV) through sexual contact [46]. The mother may then pass on EBV to the child, predisposing tumor development. However, further analysis did not show any correlation between number of self-reported lifetime sexual partners and levels of EBV titers in the mother (results not shown), nor was there an association between prevalence of maternal syphilis (as a marker of sexual behavior) and risk of Burkitt lymphoma in the child. Consequently, the significance of the findings relating to maternal sexual and reproductive history is not clear.

It is possible that some case and control children might have been misclassified, 75% of cases and 74% of controls had a histological verification of diagnosis. This is a relatively high rate of verification for an African series [16], [47]. As described previously, the jaw was the most common site for primary tumor presentation, and the mean age at diagnosis was 7.1 years, also similar to other studies [48], [49].

In conclusion, this case-control study of Burkitt lymphoma conducted among children in Malawi strengthens the evidence associating the tumor with HIV, EBV and malaria. Improved control of malaria, as well as the future development of EBV vaccines [50] could play an important role in protecting African children from this common and often fatal malignancy.

## References

[1] Parkin.Max. D, Ferlay J, Hamdi-Chérif M, Sitas F, Thomas JO, IARC (2003). Cancer in Africa: Epidemiology and Prevention;.

[2] Dzamalala C, Mdokwe C, Mkula C, Liomba N (2005). The Cancer Epidemic in Malawi: 2001–2003.

[3] Morrow RH, Gutensohn N, Smith PG (1976). Epstein-Barr virus-Malaria interaction models for Burkitt's lymphoma: Implications for preventive trials.. Cancer Research.

[4] Olweny LMC, Atine I, Kaddu-Mukasa A, Ower R, Anderson-Anvret M (1977). Epstein-Barr Virus genome studies in Burkitt's and non-Burkitt's Lymphomas in Uganda.. Journal of the National cancer Institute.

[5] Biggar RJ, Nkrumah KF (1979). Burkitt's Lymphoma in Ghana: Urban-Rural distribution, time-space clustering and seasonality.. International Journal of Cancer.

[6] Shiramizu B, Barriga F, Neequaye J, Jafri A, Dalla-Favera R (1991). Patterns of chromosomal breakpoint locations in Burkitt's lymphoma: relevance to geography and Epstein-Barr virus association.. Blood.

[7] Geser A, de Thé G, Lenoir G, Day NE, Williams EH (1982). Final case reporting from the Ugandan prospective study of the relationship between EBV and Burkitt's Lymphoma.. International Journal of Cancer.

[8] Henle G, Henle W, Clifford P, Diehl V, Kafuko G (1969). Antibodies to Epstein-Barr virus in Burkitt's lymphoma and control groups.. J Natl Cancer Inst.

[9] de Thé G, Geser A, Day NE, Tukei PM, Williams EH (1978). Epidemiological evidence for causal relationship between Epstein-Barr virus and Burkitt's lymphoma from Ugandan prospective study.. Nature.

[10] Kafuko GW, Burkitt D (1970). Burkitt's Lymphoma and Malaria.. International Journal of Cancer.

[11] Dalldorf G, Linsell C, Barnhart F, Martyn R (1964). An epidemiologic approach to the lymphomas of African children and Burkitt's lymphoma.. Perspect Biol Med.

[12] Haddow AJ, Burkitt PDenis, Wright DH (1970). Epidemiological evidence suggesting an infective element in the etiology.. Burkitt's Lymphoma.

[13] Carpenter L, Newton R, Casabonne D, Ziegler J, Mbulaiteye S (2008). Antibodies against malaria and Epstein Barr Virus in childhood Burkitt lymphoma: a case-control study in Uganda.. International Journal of Cancer.

[14] Slutsker L, Khoromana C, Hightower A, Macheso A, Wirima J (1996). Malaria infection in infancy in rural Malawi.. Am J Trop Med Hyg.

[15] Yamey G (2000). African heads of state promise action against malaria.. BMJ.

[16] Sinfield R, Banda K, Borgstein E, Broadhead R, Hesseling P (2007). Spectrum and presentation of pediatric malignancies in the HIV era: Experience from Blantyre, Malawi, 1998–2003.. Pediatric Blood & Cancer.

[17] Lazzi S, Ferrari F, Nyongo A, Palummo N, de Milito A (1998). HIV-associated malignant lymphomas in Kenya (Equatorial Africa).. Hum Pathol.

[18] Newton R, Ziegler JL, Beral V, Katongole-Mbidde E, Carpenter L (2001). A Case-Control study of Human Immunodeficiency Virus infection and cancer in adults and children residing in Kampala, Uganda.. International Journal of Cancer.

[19] Parkin MD, Garcia-Giannoli H, Raphael M, Martin A, Katongole-Mbidde E (2000). Non-Hodgkin lymphoma in Uganda: A case-control study.. AIDS.

[20] Morrow RH, Kisuule A, Mafigiri J (1974). Socioeconomic Factors in Burkitt's lymphoma.. Cancer Research.

[21] Bronzan R, Taylor T, Mwenechanya J, Tembo M, Kayira K (2007). Bacteremia in Malawian Children with Severe Malaria: Prevalence, Etiology, HIV Coinfection, and Outcome.. The Journal of Infectious Diseases.

[22] Akre O, Loren L, Steinar T, Annika L, Lars E (1999). Epstein-Barr virus and cytomegalovirus in relation to testicular-cancer risk: a nested case-control study.. International Journal of Cancer.

[23] Newton R, Carpenter L, Casabonne D, Beral V, Babiker A (2006). A prospective study of Kaposi's sarcoma-associated herpesvirus and Epstein-Barr virus in adults with human immunodeficiency virus-1.. British Journal of Cancer.

[24] Egan A, Chappel J, Burghaus P, Morris J, McBride J (1995). Serum antibodies from malaria-exposed people recognize conserved epitopes formed by the two epidermal growth factor motifs of MSP1(19), the carboxy-terminal fragment of the major merozoite surface protein of Plasmodium falciparum.. Infect Immun.

[25] Verra F, Simpore J, Warimwe GM, Tetteh KK, Howard T (2007). Haemoglobin C and S Role in Acquired Immunity against Plasmodium falciparum Malaria.. PLoS ONE e978.

[26] Microsoft (2003). Microsoft Access. 2003 ed.

[27] StataCorp (Release 2007) (2007). Intercooled Stata 9.2 for windows. Version 9.2 ed.

[28] Douek D (2003). Disrupting T-cell homeostasis: how HIV-1 infection causes disease.. AIDS Rev.

[29] Cohen JI (2000). Epstein - Barr virus Infection.. N Engl J Med.

[30] Morrow RH, Schottenfield D, Fraumeni JF (1982). Burkitt's lymphoma.. Cancer Epidemiology and Prevention.

[31] Geser A, Brubaker G, Lenoir GM, O'Conor GT, Olweny LMC (1985). A preliminary report of epidemiological studies of Burkitt's lymphoma, Epstein-Barr virus infection and malaria in North Mara, Tanzania.. Burkitt's lymphoma: A human cancer model.

[32] Geser A, Brubaker G, Draper C (1989). Effect of a Malaria suppression program on the incidence of African Burkitt's Lymphoma.. American Journal of Epidemiology.

[33] Akpogheneta OJ, Duah NO, Tetteh KKA, Dunyo S, Lanar DE (2008). Duration of Naturally Acquired Antibody Responses to Blood-Stage Plasmodium falciparum Is Age Dependent and Antigen Specific.. Infect Immun.

[34] Akenji T, Deas J (1994). Definition of populations at risk for Plasmodium falciparum infection in three endemic areas of Cameroon.. J Parasitol.

[35] Corran P, Coleman P, Riley E, Drakeley C (2007). Serology: a robust indicator of malaria transmission intensity?. Trends in Parasitology.

[36] Kinyanjui S, Conway D, Lanar D, Marsh K (2007). IgG antibody responses to Plasmodium falciparum merozoite antigens in Kenyan children have a short half-life.. Malaria Journal.

[37] Mathanga DP, Campbell CH, Taylor TE, Barlow R, Wilson ML (2005). Reduction of childhood malaria by social marketing of insecticide-treated nets: A case-control study of effectiveness in Malawi.. Am J Trop Med Hyg.

[38] Lengeler C (1998). Insecticide-treated bed nets and curtains for preventing malaria.. http://mrw.interscience.wiley.com/cochrane/clsysrev/articles/CD000363/frame.html.

[39] National Statistical Office (2004). Malawi Demographic and Health Survey 2004.. http://www.measuredhs.com/pubs/pdf/FR175/00FrontMatter.pdf.

[40] Moormann AM, Chelimo K, Sumba OP, Lutzke ML, Ploutz-Snyder R (2005). Exposure to holoendemic malaria results in elevated Epstein-Barr virus loads in children.. Journal of Infectious Diseases.

[41] Rasti N, Falk KI, Donati D, Gyan BA, Goka BQ (2005). Circulating Epstein-Barr Virus in Children Living in Malaria-Endemic Areas.. Scandinavian Journal of Immunology.

[42] Whittle H, Brown J, Marsh K, Blackman M, Jobe O (1990). The effects of Plasmodium falciparum malaria on immune control of B lymphocytes in Gambian children.. Clin Exp Immunol.

[43] Lam K, Syed N, Whittle H, Crawford D (1991). Circulating Epstein-Barr virus-carrying B cells in acute malaria.. Lancet.

[44] IARC (1997). IARC Monographs on the evaluation of carcinogenic risks to humans: Epstein Barr Virus and Kaposi's Sarcoma Herpesvirus/Human Herpesvirus 8.

[45] Mbulaiteye SM, Walters M, Engels EA, Bakaki PM, Ndugwa CM (2006). High Levels of Epstein-Barr Virus DNA in Saliva and Peripheral Blood from Ugandan Mother-Child Pairs.. J Infect Dis.

[46] Higgins CD, Swerdlow AJ, Macsween KF, Harrison N, Williams H (2007). A Study of Risk Factors for Acquisition of Epstein-Barr Virus and Its Subtypes.. J Infect Dis.

[47] Chokunonga E, Levy LM, Bassett MT, Mauchaza BG, Thomas DB (2000). Cancer Incidence in the African population of Harare, Zimbabwe: Second results from the Cancer Registry.. International Journal of Cancer.

[48] Morrow RH, Kisuule A, Pike MC, Smith PG (1976). Burkitt's Lymphoma in the Mengo districts of Uganda: Epidemiologic features and their relationship to malaria.. Journal of the National cancer Institute.

[49] Burkitt P, Denis (1958). A Sarcoma involving the Jaws in African Children.. British Journal of Surgery.

[50] Moutschen M, Leonard P, Sokal EM, Smets F, Haumont M (2007). Phase I/II studies to evaluate safety and immunogenicity of a recombinant gp350 Epstein-Barr virus vaccine in healthy adults.. Vaccine.

